# GLP-1 receptor agonists and cancer: current clinical evidence and translational opportunities for preclinical research

**DOI:** 10.1172/JCI194743

**Published:** 2025-11-03

**Authors:** Estefania Valencia-Rincón, Rajani Rai, Vishal Chandra, Elizabeth A. Wellberg

**Affiliations:** 1 Department of Pathology, College of Medicine, and; 2 Gynecologic Oncology Section, Department of Obstetrics and Gynecology, University of Oklahoma Health Sciences Center, Oklahoma City, Oklahoma, USA.; 3 Stephenson Cancer Center, Oklahoma City, Oklahoma, USA.

## Abstract

Cancer diagnoses are prevalent in people with obesity and type 2 diabetes, and abundant clinical evidence supports the protective effects of weight loss for cancer prevention. Glucagon-like peptide-1 (GLP-1) receptor agonists have revolutionized obesity and type 2 diabetes medicine and alleviate many comorbidities of these metabolic diseases. In this Review, we summarize the current clinical evidence for GLP-1 receptor agonists and cancer risk, including thyroid, pancreatic, gastrointestinal, and hormone-dependent malignancies. With few exceptions, recent meta-analyses report that GLP-1 receptor therapies do not increase cancer incidence and may lower risk in some cases. Preclinical studies reinforce the anticancer effects of GLP-1 receptor therapies, even in non-obese models. However, there are still many opportunities for translational insight as the field grows. Immune-modulating effects of GLP-1 receptor agonists are reported in several preclinical cancer studies, which may reflect direct action on immune cells or result from improved metabolic function. We highlight ongoing clinical trials for GLP-1 receptor therapies in cancer patients, and offer considerations for preclinical studies, including perspectives on the timing and duration of GLP-1 receptor agonist treatment, concurrent use of standard anticancer therapies, and interpretation of models of cancer risk versus progression.

## Introduction

In 2022, the World Health Organization estimated that more than 890 million adults live with obesity (1), defined as a body mass index (BMI) of ≥30 kg/m^2^. An additional 1.6 billion adults were classified as overweight (BMI ≥ 25 kg/m^2^) (1). Obesity is associated with comorbidities such as type 2 diabetes (T2D) and cancer (2, 3). The risk for at least 13 cancer types is elevated in people with obesity, including colon, rectum, gastric, hepatocellular, gallbladder, pancreatic, kidney, esophageal, meningioma, thyroid, endometrial, ovarian, and postmenopausal breast cancers (4). T2D and prediabetes are also more prevalent in people with obesity (5), and T2D is similarly associated with an elevated risk for several cancers, including colorectal, hepatocellular, gallbladder, pancreatic, breast, and endometrial carcinomas (6, 7). Mendelian randomization studies link genetically predicted T2D risk with the incidence of many cancers, such as pancreatic, liver, thyroid, breast, prostate, colorectal, and esophageal (6). When observational and genetic data are combined, the strongest evidence linking T2D with excess cancer risk exists for pancreatic, endometrial, and breast tumors (6). Potential tumor-promoting mechanisms common to obesity and T2D include disrupted synthesis and action of steroid hormones, elevated adipose tissue inflammation, and sustained hyperinsulinemia (7, 8). The prognosis after cancer treatment of patients with obesity or T2D is worse than that of patients who are metabolically healthy, with both obesity and T2D shortening progression-free and overall survival (7).

Agonists of the glucagon-like peptide-1 (GLP-1) receptor have emerged as leading treatments for both T2D and obesity (9). GLP-1 is a peptide product of posttranslational proteolysis of proglucagon. It is secreted into circulation by L cells in the intestine in response to nutrient intake (10), and maintains glucose homeostasis by stimulating insulin secretion, inhibiting glucagon release, slowing gastric emptying, and reducing appetite (11, 12). The actions of GLP-1 on insulin and glucagon secretion inspired the development of several GLP-1 receptor (GLP-1R) agonists that have revolutionized treatment for obesity and diabetes (12, 13). GLP-1R is expressed in pancreatic B and D cells, where it controls secretion of insulin and somatostatin, respectively (12), and is also expressed in lung, stomach, intestine, liver, kidney, heart, blood vessels, and regions in the central nervous system including the hypothalamus and brainstem (12, 14–17).

Given the widespread expression of GLP-1R and the established link between obesity and cancer, there has been a growing interest in the potential for GLP-1R agonists to modulate cancer development and progression. Here, we describe the available evidence that GLP-1R agonists may alter cancer risk, and the molecular mechanisms that may underlie the effect.

## GLP-1R agonists and cancer: clinical and preclinical evidence

GLP-1R therapies have emerged as potential effectors of cancer risk and prognosis (Table 1). Some studies using data from the FDA Adverse Event Reporting System (FAERS) reported a potential increase in cancer risk associated with GLP-1R agonist use (18, 19); however, these findings are not consistently corroborated in retrospective cohort or randomized controlled trials. In general, epidemiologic data have failed to demonstrate a persistent, excess risk for most cancers in people using GLP-1R therapies (20–25). A recent meta-analysis analyzing only tirzepatide users reported no excess risk for any cancer (breast, colon, gastric, glioblastoma, lung, lymphoma, ovarian, pancreatic, prostate, renal, skin, squamous cell, thyroid, bladder, uterine) in comparison with controls (26). In that analysis, risk for any cancer was similar between individuals with and without T2D and was lowest in patients taking the highest dose of tirzepatide. In contrast, in a large retrospective cohort study encompassing several types of cancer, Wang et al. found that compared with insulin, GLP-1R agonist use was associated with a significantly lower risk for gallbladder cancer, meningioma, pancreatic cancer, hepatocellular carcinoma, multiple myeloma, endometrial cancer, ovarian cancer, colorectal cancer, esophageal cancer, and kidney cancer (24). Compared with metformin, there was no effect of GLP-1R agonist use on cancer risk, except for kidney cancer, which was more prevalent among GLP-1R agonist recipients (24). Notably, using metformin as a control may introduce variability in risk assessments, since it is known to reduce the incidence of many cancers (27, 28). In that study, the cancer risk reduction from GLP-1R agonist use was comparable to that from lifestyle intervention (16% risk reduction; ref. 29) and bariatric surgery (32% risk reduction; ref. 30); however, some evidence suggests anticancer benefits of GLP-1R therapies beyond weight loss. A recent retrospective cohort study compared the effects of GLP-1R agonists versus bariatric surgery on the risk for obesity-associated cancers (31). Bariatric surgery caused superior weight loss, but cancer risk was 40% lower in GLP-1R agonist users than in those who had bariatric surgery, suggesting weight loss–independent anticancer mechanisms of GLP-1R therapies (31). Regarding hematologic malignancies, a retrospective cohort study investigating people receiving insulin, metformin, or GLP-1R agonists for T2D showed that GLP-1R agonist users had a significantly lower risk of myelodysplastic syndromes and myeloproliferative neoplasms compared with metformin users, and a lower risk for myeloid or lymphoid leukemia, lymphoid leukemia, non-Hodgkin lymphoma, myelodysplastic syndromes, myeloproliferative neoplasms, monoclonal gammopathy, multiple myeloma, and amyloidosis compared with insulin users (22). When these cancer subtypes were combined, GLP-1R agonist use for T2D management reduced the risk for all hematologic malignancies by 54% compared with insulin (22).

### Thyroid cancer.

Based on a range of analysis approaches and data sources, debates continue about the long-term safety of GLP-1R agonists in some patients, with thyroid cancer as one focus of the discussion (32, 33). This is in part because preclinical data show that GLP-1R agonists, including GLP-1 itself, can increase the proliferation of parafollicular C cells in the thyroid gland of rats, and in a C cell line derived from a rat medullary thyroid carcinoma (34, 35). In patients, a retrospective study based on FAERS data reported that GLP-1R agonist use was associated with an excess risk of thyroid cancer, while metformin use was not (19). However, the investigators acknowledged several limitations of their approach, including the reliance on voluntary and spontaneous reporting, lack of control groups, and the inability to infer causation (19). A nested case-control study by Bezin et al. similarly reported a greater risk for all thyroid cancers, and for medullary thyroid cancers, among current GLP-1R agonist users compared with non-users with T2D (36). Elevated risk was evident with less than 3 years of GLP-1R agonist exposure, which could mean that these drugs promote progression of existing precancerous lesions. This case-control study generated several commentary articles, each urging caution when concluding that GLP-1R agonists are tumor-promoting in the thyroid (37–39). Noted concerns included detection bias, meaning that patients using GLP-1R agonists may be frequently monitored by physicians and more likely to be diagnosed with cancer (37). In addition, obesity, which increases thyroid cancer risk (40), was more prevalent in cases compared with controls (37). Patients with thyroid nodules or cancer were not excluded before beginning GLP-1R therapy, which might influence tumor diagnosis in patients after treatment (38). Nonetheless, the FDA issued a warning for most GLP-1R–targeted therapies for patients with a history of medullary thyroid carcinoma or multiple endocrine neoplasia syndrome, which remains in place today (41).

A 2012 meta-analysis reported no significant increase in thyroid cancer risk in people using liraglutide compared with placebo, insulin, or other antihyperglycemic drugs (42). These findings were corroborated 10 years later in a meta-analysis of randomized controlled trials published by Hu et al. (43). The risk for cancer was not related to the patient’s underlying condition (T1D, T2D, overweight/obesity) or influenced by the control (placebo, insulin, or another antihyperglycemic drug) (43). In contrast to this study, a meta-analysis by Silverii et al. reported that GLP-1R therapy use was associated with a significantly greater risk for overall thyroid cancer in a fixed-effect analysis (44); however, a fragility index of 1 was noted, meaning that it would take only one additional case of thyroid cancer in the comparator group for the association to lose significance. When a random-effect model was applied, the association between GLP-1R therapy use and thyroid cancer was not significant (44). Nagendra et al. (45) highlighted key differences across the studies analyzed above, emphasizing that some of them mainly focused on the first-generation GLP-1R agonists exenatide, liraglutide, and dulaglutide, with little or no inclusion of people taking semaglutide. In that analysis, semaglutide treatment did not increase the risk for thyroid or pancreatic cancers, nor for other neoplasms, whether it was compared with placebo or other antihyperglycemic drugs (45). Following the Silverii study was a meta-analysis that reported a 62% excess risk for thyroid cancer after GLP-1R agonist exposure (46). The authors suggested a potentially causal relationship; however, subgroup analyses were not performed on individual drugs.

In preclinical thyroid cancer models (47), semaglutide was shown to have tumor-suppressive effects through actions on immune cells. Semaglutide treatment had no direct inhibitory effects on proliferation of human papillary thyroid carcinoma cells in vitro, but it suppressed the growth and proliferation of human thyroid cancer cells grafted into nude mice. In that study, semaglutide was initiated after palpable tumors formed, and mice were not fed a high-fat diet to induce obesity. Although the mice were immune deficient, semaglutide treatment shifted macrophage polarization from M2 to M1 phenotypes, consistent with its tumor-suppressive effects. The influence of semaglutide on macrophages was demonstrated to be direct and to occur through suppression of PPARγ, as these effects were seen in cultured immune cells. The clinical and preclinical evidence showed that although first-generation GLP-1 analogs might be associated with increased thyroid cancer risk, semaglutide might instead have protective properties.

### Pancreatic cancer.

As with thyroid cancer, GLP-1R agonist use has been linked to pancreatic cancer risk, although without clear consensus on the direction. In 2011, an observational study using FAERS data (32) reported an elevated risk for pancreatic cancer in people using exenatide compared with other medications not indicated for diabetes, which prompted an FDA warning (48). The conclusions of later cohort studies or meta-analyses were inconsistent, reporting either no excess risk or an elevated pancreatic cancer risk associated with GLP-1R therapies (49–51); however, the risk may be influenced by the severity of T2D (50). One meta-analysis reported no excess risk for pancreatic cancer in patients with T2D using GLP-1R agonists compared with placebo or other antihyperglycemic drugs (52). A recent retrospective cohort study similarly showed that GLP-1R agonist use was associated with a lower risk of pancreatic cancer compared with use of several other antihyperglycemic drugs, including insulin, metformin, DPP4 inhibitors, SGLT2 inhibitors, sulfonylurea, or thiazolidinediones (TZDs) (53). The reduction in pancreatic cancer risk was greater in patients with obesity compared with those without. Patients receiving combination therapy that included GLP-1R agonists had a lower risk for pancreatic cancer when compared with those on single, non–GLP-1R therapy, but not when the combination therapy was compared with GLP-1R monotherapy (53), suggesting a specific antitumor effect of GLP-1R activation. In that study, the combination of GLP-1R therapy and insulin predicted a greater risk for pancreatic cancer compared with GLP-1R monotherapy, consistent with a reported tumor-promoting role of insulin in preclinical pancreatic cancer models (54–56). Notably, patients receiving insulin for glucose control likely have more advanced T2D, which could influence tumor progression irrespective of antiglycemic medication.

A recent preclinical study by Marathe et al. evaluated pancreatic cancer endpoints in a mouse model of obesity (57). Adult male mice were given a high-fat diet and assigned to receive semaglutide, retatrutide (GLP-1R/GIPR/GCGR triple agonist), dietary calorie restriction, or vehicle control before implantation of pancreatic cancer cells. Compared with control mice fed an ad libitum high-fat diet, those receiving retatrutide, semaglutide, or calorie restriction lost significant weight, with the greatest weight loss seen in retatrutide-treated mice. Retatrutide improved body composition by reducing mass of epididymal adipose — a visceral depot in mice — while semaglutide and calorie restriction had no effect on this measure. Each intervention improved glucose tolerance. The proportion of mice with palpable tumors was lower with retatrutide, but was not substantially impacted by semaglutide or calorie restriction. The growth rate and volume of established tumors were lower in each intervention group compared with the ad libitum control, with retatrutide treatment having the greatest antitumor effect. In another cohort, retatrutide was discontinued at the time of pancreatic cancer cell implantation. This withdrawal led to weight and adipose regain, a slight increase in fasting blood glucose, and loss of the inhibitory effect of retatrutide on tumor engraftment. However, the growth of the established tumors continued to be suppressed in retatrutide-withdrawn animals. This study suggests that the mechanisms that drive the initial outgrowth of tumors from a small number of cells may not be the same as those driving the rapid growth of established tumors. Within the tumors, retatrutide increased expression of genes associated with inflammation and antitumor immunity and decreased expression of genes associated with metabolism, changes that were reversed upon retatrutide withdrawal. The investigators performed a similar experiment in obese mice with lung adenocarcinoma (57), which is not considered an obesity-associated cancer. Again, retatrutide reduced tumor engraftment rate, delayed tumor onset, and suppressed tumor growth. Consistent with data from the pancreatic cancer model, retatrutide treatment induced systemic immune reprogramming by reducing circulating CD11b^+^ cells, macrophages, and monocytic myeloid-derived suppressor cells, and increasing PD-1 expression in CD8^+^ T cells. Effects of semaglutide or calorie restriction on the immune environment were not reported (57).

Cencioni et al. investigated the impact of semaglutide on the tumor microenvironment of obesity-associated pancreatic ductal adenocarcinoma (58). In that study, male mice were given a low- or high-fat diet and then received pancreatic cancer cells, similar to the model described in Marathe et al. (57). The high-fat diet accelerated tumor growth, increased collagen deposition in the tumor microenvironment, and reduced the infiltration of CD3^+^ immune cells. Semaglutide administered 1 week before pancreatic cancer organoid implantation did not impact engraftment rate but did slow growth and development of advanced lesions. This was accompanied by a reduced mesenchymal stromal cell signature and collagen deposition, facilitating increased infiltration of T lymphocytes into pancreatic tumors (58). Thus, while GLP-1R agonist treatment suppresses tumors in these models, the triple agonist (retatrutide) may have a more prominent, and potentially durable, antitumor effect in the context of obesity.

### Gastrointestinal cancers.

Clinical studies consistently demonstrate a protective effect of GLP-1R agonists against several types of gastrointestinal cancers. A recent meta-analysis covering 90 studies found no excess risk for hepatic, biliary tract, pancreatic, colorectal, or gallbladder cancers in people using GLP-1R therapies (59). In that analysis, studies included patients who were prescribed the drugs for T2D or for weight loss. No excess risk for any gastrointestinal cancer was seen when analyses were restricted to placebo-controlled trials or when the dose of GLP-1R agonist was considered (59). A meta-analysis by Pasta et al., which included five studies, found that GLP-1R therapies reduced hepatocellular carcinoma risk by 58%, compared with insulin or other antihyperglycemic drugs (60). A prior meta-analysis of seven studies, four of which were analyzed by Pasta et al., showed a similar reduction in hepatocellular carcinoma risk in GLP-1R agonist users with T2D compared with those using insulin or other antihyperglycemic drugs (61). In a meta-analysis of five studies that included over 2 million people, colorectal cancer risk was significantly lower in people using GLP-1R agonists compared with those using TZDs, SGLT2 inhibitors, and insulin, and was equivalent to that in people using sulfonylureas, metformin, or DPP4 inhibitors (23). The use of exenatide, liraglutide, or semaglutide was recently shown to associate with an 18% lower risk for colorectal cancer compared with TZDs and 43% lower risk compared with insulin (23). Insulin therapy is associated with a 69% greater risk for colorectal cancer, which may result from the mitogenic and pro-survival effects of insulin on colorectal cancer cells (62).

Preclinical studies of gastrointestinal cancers reinforce the tumor-suppressive effects of GLP-1R therapies. In female C57BL/6 mice grafted with MC38 colon cancer cells, treatment with exendin-9 for 2 weeks after cancer cell implantation attenuated tumor growth (63). Compared with controls, tumors from exendin-9–treated mice had more infiltrating CD3^+^ T cells and CD8^+^ effector memory T cells (63), supporting a role for GLP-1R activation in modulating the tumor immune environment. Similarly, exenatide was reported to inhibit tumor growth, vascularization, inflammation, and cancer cell proliferation in a mouse model of diabetes and chemical carcinogen–induced colon cancer (64). In that study, diabetes was induced by treatment of high fat/sucrose–fed male mice with streptozotocin. Intervention with the chemotherapeutic agent cisplatin or exenatide began alongside tumor initiation with 1,2-dimethylhydrazine, allowing the investigators to study the tumor-preventive effects of these drugs in immune-competent mice (64). In a model of cholangiocarcinoma, a cancer of the bile duct, liraglutide did not affect cell proliferation in vitro, but did moderately inhibit cell migration (65). Male immune-compromised mice were grafted with human cholangiocarcinoma cells, and liraglutide treatment was initiated after palpable tumor formation. In this setting, liraglutide treatment inhibited tumor growth concomitant with lower activation of STAT3, MAPK, and Akt pathways. In that study, the mice were not fed a high-fat diet, and body mass was not reported (65). In another investigation using C57BL/6 male mice, liraglutide treatment suppressed growth of grafted hepatocellular carcinoma tumors (66). Liraglutide was initiated after measurable tumors had formed, and inhibitory effects on tumor growth were evident by 3 days of treatment. Liraglutide treatment increased IFN-γ–producing cells in tumors, and the antitumor effect of the drug was reversed by inhibition of natural killer cells, but not CD8^+^ T cells (66). Overall, evidence suggests the potential for GLP-1R agonists to lower the risk for gastrointestinal cancers, even in the absence of obesity. Further work is needed to determine whether these effects are also seen in females and whether they are reproducible with newer-generation drugs.

### Hormone-associated cancers.

Steroid hormones are influenced by adiposity, and accordingly, cancers that are hormone driven, such as those of prostate, endometrium, and breast, have been linked to obesity. GLP-1R agonist use consistently associates with a lower risk for prostate cancer in clinical studies. Multiple recent meta-analyses covering studies that included a variety of controls, such as metformin, insulin, DPP4 inhibitors, SGLT2 inhibitors, sulfonylureas, and TZDs, demonstrated a reduction in prostate cancer incidence in men with T2D who took GLP-1R therapies (67–69). Whether this attenuation of risk was due to weight loss, improved insulin sensitivity, or another mechanism is unclear. Prostate cancer risk is not strongly associated with T2D. In fact, men with T2D have lower levels of prostate-specific antigen (PSA), which remains the standard for prostate cancer risk assessment (70, 71). Notably, a high waist-to-hip ratio or waist circumference, each of which reflects visceral adiposity, independently predicts elevated prostate cancer risk (71, 72). Hyperinsulinemia is also a risk factor for prostate cancer (73, 74), and may occur separately from obesity or years before T2D diagnosis (75).

Apart from the meta-analyses described above that included gynecologic malignancies among multiple cancers (24, 26), evidence to support a role for GLP-1R therapies in lowering endometrial cancer risk or progression is limited to preclinical studies and corroboration of mechanisms in human specimens (76). In general, GLP-1R agonists may attenuate endometrial cancer growth and tumor progression, potentially through increasing the sensitivity of cells to progesterone (77), which is a standard antiproliferative treatment for endometrial hyperplasia. A recent preclinical study evaluated the therapeutic efficacy of tirzepatide in a transgenic mouse model of endometrial cancer under high-fat diet–induced obese and low-fat-diet lean conditions (78). In that study, tirzepatide treatment began 8 weeks after Cre-mediated excision of *Lkb1* and *Trp53* in the uterine horns, which initiates endometrial cancer. Four weeks of tirzepatide treatment significantly decreased body mass and tumor masses in obese and lean mice. In both diet groups, tumor cell levels of the proliferation marker Ki67 and the anti-apoptotic marker Bcl-xL were lower with tirzepatide treatment. Tirzepatide improved metabolic and inflammatory environments, though the specific effects differed between obese and lean mice. In the obese group, tirzepatide downregulated genes related to ErbB signaling and glycolysis/gluconeogenesis pathways, while increasing genes related to fatty acid degradation, cortisol synthesis/secretion, and B cell receptor signaling. In lean mice, tirzepatide downregulated genes related to phospholipase D signaling, eicosanoids, and pentose and amino sugars, and increased endocannabinoid-related genes. GLP-1R expression, measured by RNA sequencing, was greater in lean mice compared with obese mice and was lower after tirzepatide treatment (78). These results suggest a promising beneficial effect of GLP-1R agonists on endometrial cancer risk and on progression, even in the absence of obesity, although much remains to be investigated, especially regarding the potential of combining GLP-1R agonists with standard therapies (76).

Large retrospective studies demonstrate that the risk for breast cancer is not elevated in people using GLP-1R agonists, particularly with follow-up of more than 2 years (21, 24, 79), nor is there excess risk for benign or premalignant breast neoplasms (21). One study found a greater risk of breast cancer diagnosis in patients using GLP-1R therapies who had no prior history of mammography screening, but not in those who had a history of regular mammograms. That study also demonstrated that mammography screening rates were higher in patients using GLP-1R therapies compared with controls (79). Therefore, the newly diagnosed breast cancers may reflect increased screening. An independent study reported excess breast cancer diagnoses that increased with weight loss categories (80). Patients who lost more than 10% weight were most likely to be diagnosed with breast cancer. In that study, referrals for diagnostic breast imaging and mammography screening also increased with increasing weight loss category (80), again suggesting a better ability to diagnose breast cancer in women taking GLP-1R agonists.

A recent preclinical study investigated the efficacy of tirzepatide compared with chronic calorie restriction in a xenograft model of obesity-associated breast cancer (81). Diet-induced obese female mice were randomized to remain on their diet and receive tirzepatide or vehicle, or they were switched to a calorie-restricted diet for weight loss. The low-fat-fed lean mice did not receive weight loss interventions. Eight weeks after calorie restriction or tirzepatide treatments, mice were orthotopically injected with triple-negative E0771 breast cancer cells, and tumor growth was monitored. Tirzepatide treatment promoted weight loss, improved glycemic control, and suppressed mammary tumor growth. Chronic calorie restriction caused more weight loss than tirzepatide, and had a greater tumor-suppressive effect. Tumor mass directly correlated with body weight, with each 5 g increase in whole-body mass predicting a 0.7 g increase in tumor mass. Importantly, glucagon levels were significantly higher in calorie-restricted mice compared with those given tirzepatide, suggesting that triple agonists targeting the glucagon receptor might elicit a superior antitumor effect, more like chronic calorie restriction. In that study, the investigators noted that they maintained the tirzepatide regimen after tumor implantation to prevent the rapid weight gain that accompanies discontinuation of GLP-1R agonist therapy (81). This point is important in considering how preclinical studies will translate to new practice guidelines. In the clinical setting, once a patient is diagnosed and cancer treatment begins, physicians might choose to stop GLP-1R therapy, which could indirectly promote breast cancer progression through weight regain.

Stanisavljevic et al. investigated the effect of semaglutide on triple-negative 4T1 mammary cancer cells in immune-competent, lean BALB/c mice (82). There, semaglutide treatment was initiated after engraftment of cancer cells. Semaglutide significantly delayed the emergence of tumors and reduced tumor size and metastatic spread to the liver, without affecting tumor vascularization. In cultured cancer cells, semaglutide was not cytotoxic, suggesting an indirect antitumor mechanism. In vivo, CD11c^+^ dendritic cells were elevated and FoxP3^+^IL-10^+^ T cells were lower in the spleens and primary tumors of semaglutide-treated mice compared with controls. Semaglutide also promoted tumor T cell infiltration and increased the proportion of CD45^+^CD8^+^ cells. The in vivo tumor-suppressive effect of semaglutide remained when NK cells were depleted, but was eliminated upon CD8^+^ cell depletion, suggesting a role for adaptive immune cells in mediating the effect of GLP-1R activation. Along these lines, treatment of isolated splenocytes with semaglutide increased the percentage of cells expressing IL-1β, TNF-α, IFN-γ, and IL-10. This specific study supports multiple plausible mechanisms associated with the inhibitory effect of GLP-1R agonists on breast tumor progression (82), strongly suggesting the potential to enhance immune-modulating therapies. Together, these studies indicate that GLP-1R therapies inhibit breast cancer growth, potentially through mechanisms unrelated to weight loss or obesity.

## Tumor-suppressive mechanisms of GLP-1R agonists

GLP-1R agonists are proposed to influence cancer risk and progression through multiple mechanisms, both indirectly and through direct action on cancer cells themselves (83). However, many of the mechanistic studies have relied on imprecise reagents, which might yield results that are not reproducible in independent investigations. Accurate detection of GLP-1R transcript and protein can be challenging (84, 85), and its low abundance requires caution in interpreting data on receptor localization and distribution (14, 17, 86). Ast et al. recently described common pitfalls encountered with GLP-1R detection and provided comprehensive guidelines for antibody selection and appropriate positive and negative controls depending on the species, tissue, and experiment type (86).

**Figure 1:**
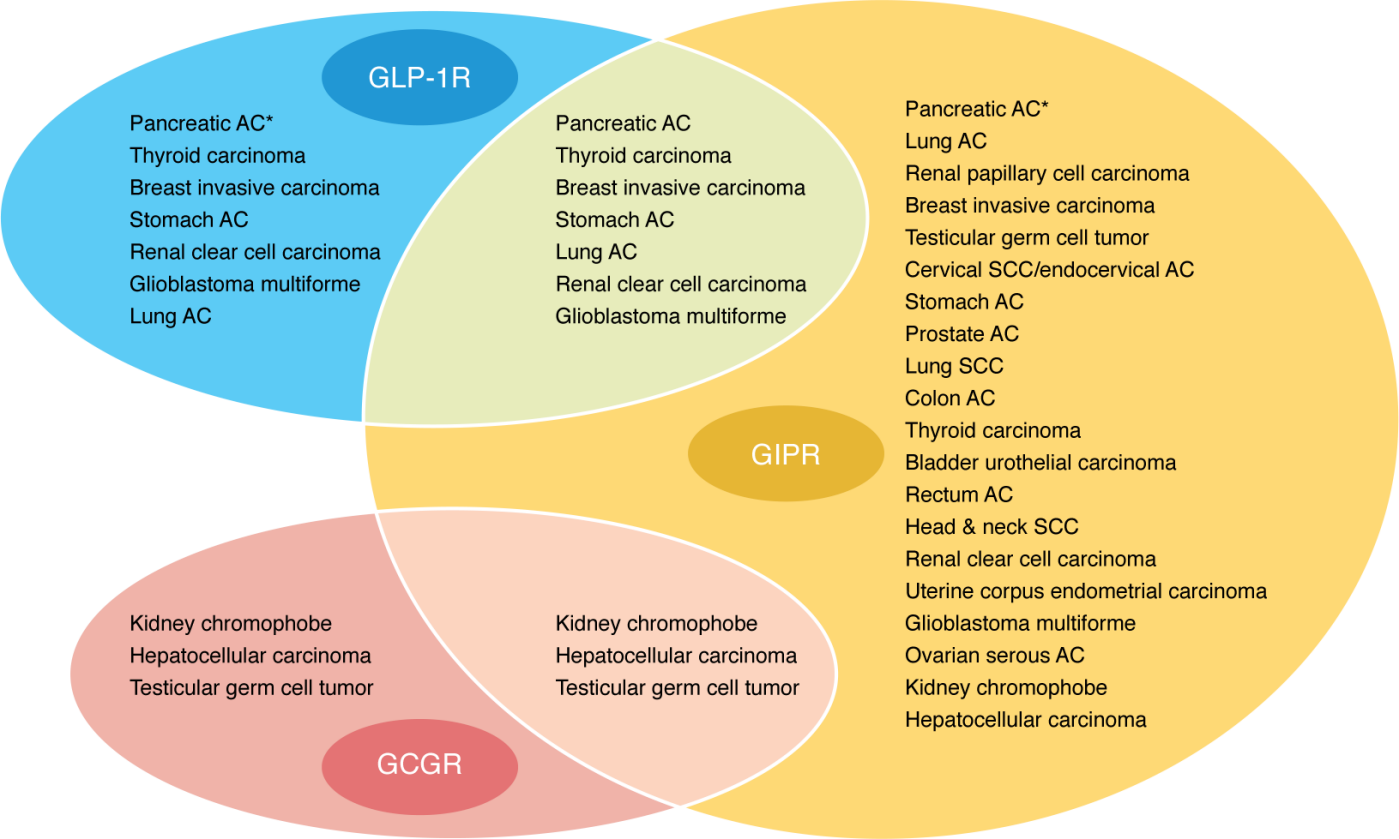
Expression of GLP-1R, GIPR, and GCGR in human tumor tissues. Tumor tissues with median expression of GLP-1R, GIPR, or GCGR greater than 0 pTPM (transcripts per million for protein-coding genes) were ranked in descending order of receptor expression. Tissues in which two receptors were expressed are indicated in the intersections for those receptors. No tissues demonstrated expression of all three receptors. Asterisks indicate tissue enhanced expression. AC, adenocarcinoma; SCC, squamous cell carcinoma. Tumor gene expression is available from the Human Protein Atlas (www.proteinatlas.org) (118); August 2025.

GLP-1R mRNA is detected in some cancers, including pancreatic, thyroid, and invasive breast tumor tissues (Figure 1). Comparatively, GIPR, which is targeted by dual agonists (e.g., tirzepatide), is expressed in many cancers, while the glucagon receptor (GCGR), which is targeted by triple agonists (e.g., retatrutide), is only detected in a few cancer types (Figure 1). We speculate that drugs that target both GLP-1R and GIPR might have better effectiveness against cancers with high expression of both receptors, such as pancreatic, thyroid, breast, and lung cancers. GLP-1R expression has been associated with the prognosis of patients with varying malignancies, but the direction of association depends on the cancer type (87). High GLP-1R levels in tumors predicted longer overall survival of patients with bladder, breast, esophageal adenocarcinoma, renal clear cell, and thyroid cancers. In contrast, patients with high tumor GLP-1R expression in squamous cell carcinomas of cervix or lung, or endometrial carcinoma, had a relatively poor prognosis. GLP-1R expression was not related to the prognosis of patients with esophageal squamous cell carcinoma, colon cancer, hepatocellular carcinoma, ovarian cancer, or pancreatic cancer (87). These differences in patient outcomes are likely influenced by cancer etiology, treatment regimen, follow-up interval, and other underlying factors such as patient BMI or metabolic function. One limitation of evaluating tumor gene expression of GLP-1R is that many public datasets interrogate tumor core biopsies, which provide samples that contain fibroblasts, immune cells, and vascular cells in addition to malignant cells. Defining the cellular targets of GLP-1R therapies in heterogeneous tumors will clarify their preventive or therapeutic mechanisms, which may also intersect with the metabolic and inflammatory pathways linked to obesity.

In addition to controlling glucose homeostasis, some GLP-1R therapies are used to treat obesity (9). Aside from BMI, adult weight gain is an independent cancer risk factor (88–92). Adipose tissue expansion during weight gain is accompanied by the production of growth factors and cytokines, many of which are implicated in cancer incidence or progression (93–100). Engorged adipocytes recruit immune cells, increasing local and systemic levels of proinflammatory factors, such as TNF-α, IL-6, and IL-1β (101, 102). Conversely, weight loss, whether through lifestyle changes or surgical interventions, lowers the risk for some obesity-associated cancers in men and women (88, 103), including breast cancer (104–107) and endometrial cancer (107). A common theme emerging from the preclinical studies of GLP-1R agonists and cancer is the potential to promote antitumor immune cell phenotypes (47, 57, 58, 66, 82), including in lean mice that were not given a high-fat diet (47, 66, 82). The effect of GLP-1R agonists in this setting may occur through direct action of these drugs on immune cells themselves, but studies are lacking to demonstrate immune cell–autonomous mechanisms of GLP-1R action in tumors in vivo.

Another tumor-suppressive effect of GLP-1R agonists might be the ability to reduce basal hyperinsulinemia (103, 104). Genetically predicted insulin concentrations are directly associated with risk for endometrial, pancreatic, and breast cancers (6), and obesity, diabetes, and prediabetes each associate with basal and postprandial hyperinsulinemia (108–110). Insulin is a crucial regulator of nutrient metabolism in adipocytes, muscle, and liver, but also has mitogenic effects in cancer cells. In contrast to glucose or hemoglobin A1c, fasting insulin is not routinely measured in the clinic, but hyperinsulinemia can occur years before glucose is chronically elevated (75). Some studies document reductions in fasting insulin levels with GLP-1R therapy in cancer settings (57, 69, 81, 111); however, insulin levels are not always reported. The benefit of GLP-1R agonists for reducing chronic hyperinsulinemia might be one mechanism that explains the inhibitory effects on cancer risk in clinical studies and on cancer progression in preclinical settings.

### Distinguishing risk and progression in preclinical studies.

To date, almost all clinical studies on GLP-1R therapies and cancer describe risk as the primary outcome. As described recently (112), there is the potential for bias in methodology of some clinical trials, including a lack of available data about use of these drugs in people without T2D or obesity. Risk, or incidence, refers to the development or diagnosis of cancer, which is influenced by several factors, including genetic predisposition, environmental exposure, and lifestyle. In contrast, progression refers to the recurrence or continued growth of established or metastatic tumors, often after treatment. Mouse models of cancer commonly rely on subcutaneous or orthotopic engraftment of malignant cells. The proportion of mice that grow tumors, the tumor growth rate, and metastatic progression can be evaluated for many cancer types. These studies might more closely model progression than risk, since tumors grow from a small number of established cancer cells, much like local recurrence of a tumor after incomplete surgical resection or pathologic response. The mechanisms driving malignant transformation of normal cells and initial tumor growth are potentially distinct from those that influence the recurrence of a tumor at the primary site or the outgrowth of a distant organ metastasis (113). Therefore, in preclinical models the effect of GLP-1R therapy should be investigated in studies of early malignant transformation and primary tumor development to better align with current clinical evidence.

In clinical studies, there is growing need for trials that enroll patients being treated for cancer to investigate the effects of GLP-1R treatments in these unique populations (114). Data presented at the American Society of Clinical Oncology annual meeting indicate that patients who take GLP-1R therapies during breast cancer treatment can achieve clinically meaningful weight loss (115–117), which may improve their prognosis. Several planned or ongoing clinical trials will address GLP-1R therapy use in people diagnosed with cancer. An early phase I trial will determine whether tirzepatide and semaglutide are practical and effective for weight management and glucose control in women with endometrial cancer taking chemotherapy, although cancer-related endpoints are not included (ClinicalTrials.gov NCT06751589). In a different trial (NCT06518837), tirzepatide will be evaluated in patients during adjuvant treatment for breast cancers that are hormone receptor (HR) positive and human epidermal growth factor receptor 2 (HER2) negative, the most prevalent breast cancer subtype. The primary outcome of this trial is the proportion of patients who achieve 5% or greater weight loss during their cancer treatment, but invasive breast cancer–free survival and distant relapse–free survival will also be determined over 3 years. A similar trial (NCT06517212) will focus primarily on breast cancer–related outcomes, aiming to determine whether tirzepatide-induced weight loss prevents the development of metastatic disease and improves disease-free survival in patients with HR-positive, HER2-negative high-risk breast cancer. In men with prostate cancer, the safety and tolerability as well as metabolic effects of semaglutide will be evaluated during androgen deprivation therapy; however, this study does not include cancer-specific endpoints (NCT06908694). These planned or ongoing clinical trials will provide important resources to validate potential mechanisms identified in preclinical studies of GLP-1R therapies, where cancer progression is often the primary endpoint.

## Conclusion

Evidence from numerous clinical studies shows the ability of GLP-1R agonists to reduce cancer risk in many people with T2D or obesity, but most studies have not yet investigated how these therapies affect cancer risk in people without metabolic diseases, nor whether GLP-1R therapies will reduce cancer-specific mortality. Preclinical studies also demonstrate anticancer effects of contemporary GLP-1R–targeted drugs even in the absence of obesity. It is not known whether the same mechanisms are responsible for the reduction in cancer incidence and for suppression of established cancer growth, but emerging evidence points to alterations in immune cell phenotypes and/or reduction in circulating insulin that accompanies improved glucose tolerance. New clinical trials will evaluate the effectiveness of GLP-1R therapies in the context of currently approved cancer treatments, which might reveal unique mechanisms of drug action and vulnerabilities of cancer cells at different stages of disease. In laboratory studies, relevant considerations include the timing of GLP-1R therapy administration relative to when tumors form, whether the treatments are combined with standard therapies as they would be in clinical trials, and the underlying metabolic condition of the animals. The potential exists for GLP-1R–targeted therapies to substantially reduce the global burden of obesity-associated diseases, including cancer.

## Figures and Tables

**Table 1:**
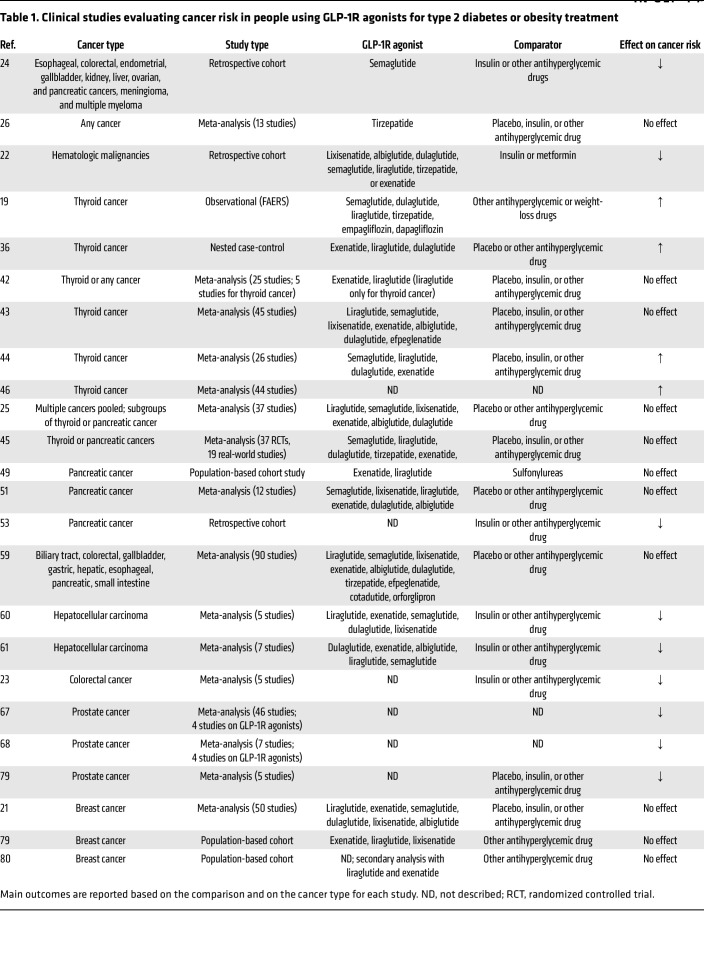
Clinical studies evaluating cancer risk in people using GLP-1R agonists for type 2 diabetes or obesity treatment

## Version 1 11/03/2025

Electronic publication
